# The relationship between tumour dosimetry, response, and overall survival in patients with unresectable Neuroendocrine Neoplasms (NEN) treated with ^177^Lu DOTATATE (LuTate)

**DOI:** 10.1007/s00259-023-06257-6

**Published:** 2023-05-15

**Authors:** R. Alipour, P. Jackson, M. Bressel, A. Hogg, J. Callahan, R. J. Hicks, G. Kong

**Affiliations:** 1 Department of Cancer Imaging, Peter MacCallum Cancer Centre, Melbourne, Australia; 2 The Sir Peter MacCallum Department of Oncology, The University of Melbourne, Melbourne, Australia; 3 Centre for Biostatistics and Clinical Trials, Peter MacCallum Cancer Centre, Melbourne, Australia; 4 Department of Medicine, St Vincent’s Medical School, The University of Melbourne, Melbourne, Australia

**Keywords:** Radiopharmaceutical Dosimetry, ^177^Lu DOTATATE (LuTate) therapy, Peptide Receptor Radionuclide Therapy (PRRT), Gastro-entero-pancreatic neuroendocrine neoplasm GEP NEN, Radiosensitising Chemotherapy

## Abstract

**Abstract:**

Peptide Receptor Radionuclide Therapy (PRRT) delivers targeted radiation to Somatostatin Receptor (SSR) expressing Neuroendocrine Neoplasms (NEN). We sought to assess the predictive and prognostic implications of tumour dosimetry with respect to response by ^68^ Ga DOTATATE (GaTate) PET/CT molecular imaging tumour volume of SSR (MITV_SSR_) change and RECIST 1.1, and overall survival (OS).

**Methods:**

Patients with gastro-entero-pancreatic (GEP) NEN who received LuTate followed by quantitative SPECT/CT (Q-SPECT/CT) the next day (Jul 2010 to Jan 2019) were retrospectively reviewed. Single time-point (STP) lesional dosimetry was performed for each cycle using population-based pharmacokinetic modelling. MITV_SSR_ and RECIST 1.1 were measured at 3-months post PRRT.

**Results:**

Median of 4 PRRT cycles were administered to 90 patients (range 2–5 cycles; mean 27.4 GBq cumulative activity; mean 7.6 GBq per cycle). 68% received at least one cycle with radiosensitising chemotherapy (RSC). RECIST 1.1 partial response was 24%, with 70% stable and 7% progressive disease. Cycle 1 radiation dose in measurable lesions was associated with local response (odds ratio 1.5 per 50 Gy [95% CI: 1.1–2.0], *p =* 0.002) when adjusted by tumour grade and RSC. Median change in MITV_SSR_ was -63% (interquartile range -84 to -29), with no correlation with radiation dose to the most avid lesion on univariable or multivariant analyses (5.6 per 10 Gy [95% CI: -1.6, 12.8], *p =* 0.133). OS at 5-years was 68% (95% CI: 56–78%). Neither baseline MITV_SSR_ (hazard ratio 1.1 [95% CI: 1.0, 1.2], *p =* 0.128) nor change in baseline MITV_SSR_ (hazard ratio 1.0 [95% CI: 1.0, 1.1], *p =* 0.223) were associated with OS when adjusted by tumour grade and RSC but RSC was (95% CI: 0.2, 0.8, *p =* 0.012).

**Conclusion:**

Radiation dose to tumour during PRRT was predictive of radiologic response but not survival. Survival outcomes may relate to other biological factors. There was no evidence that MITV_SSR_ change was associated with OS, but a larger study is needed.

**Supplementary information:**

The online version contains supplementary material available at 10.1007/s00259-023-06257-6.

## Introduction

Peptide receptor radionuclide therapy (PRRT) delivers targeted systemic radiotherapy through radionuclide delivery (^111^In, ^90^Y, ^177^Lu and several other particle emitting radioisotopes) to tumour cells which highly express somatostatin receptors (SSR). PRRT has shown promise in the treatment of advanced well-differentiated neuroendocrine neoplasia (NEN), and since its introduction in the early 1990s has undergone progressive clinical validation through the 2000s [1–5]. NEN represents a heterogeneous group of tumours arising from the diffuse endocrine system, most typically from gastro-entero-pancreatic (GEP) origins as well as the bronchopulmonary system and thymus. The World Health Organization (WHO) currently recognizes 4 main histopathological subgroups with strong prognostic implications: G1 (Ki-67 < 3%), G2 (Ki-67 3–20%), and well-differentiated G3 (Ki-67 > 20%) neuroendocrine tumor (NET), which are progressively more aggressive, and poorly differentiated neuroendocrine carcinoma (NEC), which is the most aggressive subset. Despite the low incidence of each individual subtype, the total number of new diagnoses of NEN is increasing worldwide [6, 7].

The NETTER-1 trial (a prospective randomised phase 3 study of ^177^Lu-DOTATATE in G1 and low G2 midgut NEN using a fixed administered activity for 4 treatment cycles) has confirmed efficacy of PRRT with improved progression free survival (PFS), objective response rate (ORR) and quality of life (QOL) parameters compared to high-dose long-acting repeatable (LAR) octreotide injections [8]. The final long-term follow up of NETTER-1 trial showed 11.7 months increase in overall survival (OS) in PRRT arm compared to the control arm; however, this was not statistically significant, which was thought to relate to the high cross-over rate between the two arms [9]. The results from this trial together with prior data from many other institutional series have led to the regulatory approval of ^177^Lu-DOTA-octreotate (LuTathera®, Advanced Accelerator Applications) in the USA and some countries in Europe for patients with progressive metastatic G1 and G2 GEP NET [10, 11].

An open question with respect to PRRT is whether individual dosimetry predicts response, and whether it may help to personalize treatment and improve outcomes compared to protocols with a fixed administered activity for each cycle. Most oncological therapies have dose-dependent responses that have been established by dose escalation studies in which dose limitations are based primarily on toxicity in normal tissues but aim to maximize exposure to tumour sites. With respect to physical radiation dose delivery, relatively accurate measures of tumour and normal tissue radiation doses are possible and dose planning and verification can be rigorously applied. Radionuclide therapy is generally prescribed as an administered activity, usually in GBq or mCi, however the efficiency of radiation absorbed dose delivery (Gy per GBq) is specific to an individual’s anatomy. While there is likely to be a relationship between administered activity and radiation dose to tumour and normal tissues, the reality is that the uptake and residence time in tissues, expressed as cumulative activity, are critical to the radiation dose absorbed.

Only a limited number of studies have directly assessed radiation dosimetry to tumour and its effect on tumour size response in NEN patients treated with PRRT. A prospective observational study of 200 patients with advanced metastatic NEN showed patients in whom the absorbed dose to normal kidneys reached 23 Gy had a longer OS than those in whom it did not [12]. This study however did not measure the direct dosimetry to the tumour. Initial results from a personalized PRRT (*P*-PRRT) trial showed a median 1.26-fold increase in the cumulative maximum tumour absorbed dose with promising response rates and favourable tolerance profile compared to a fixed administered activity [13]. A prospective study of 24 patients with metastatic pancreatic NET treated with ^177^Lu-DOTATATE showed a dose-response relationship with significant correlation between tumour absorbed dose and tumour size reduction on a single lesion basis in lesions greater than 2.2 cm [14]. A small retrospective study of 13 patients treated with ^90^Y-DOTATATE also showed significant correlation between tumour absorbed dose and tumour size reduction [15].

Image-based dosimetry calculation in radionuclide therapy is a complex field and one of the major challenges limiting available data is the need to improve methodology and develop quantitative imaging protocols using accessible techniques and equipment. Recent research has led to significant improvements in this field, including the establishment of automated voxel dosimetry software [16, 17]. This image-based protocol methodology allows for dose to tumour and physiologic organs to be calculated during the PRRT cycles in a consistent and reproducible manner. Based on a prospective evaluation of multiple timepoint dosimetry, a pharmacokinetic model has been developed that allows single time-point evaluation of tumour dose to be performed with appreciation of introduced uncertainties. Although imaging at 24-h may not provide the most accurate estimate of time-integrated (cumulative) activity [18], this methodology was chosen as a pragmatic balance between accuracy and the feasibility to derive meaningful quantitative data from routine practice.

PRRT is generally well tolerated with limited acute and medium-term toxicity profiles suggesting that further escalation of administered activity in single cycles or over the course of treatment could be safely delivered. The main concerns include potential renal and marrow toxicities. A recent large series of 807 patients found that severe renal complications were uncommon (1.5%) [19]. A prospective study of 323 patients treated with ^177^Lu-DOTATATE, in whom 228 patients had a mean absorbed dose to the kidneys of 20 ± 5 Gy, showed no correlation between creatinine clearance loss and kidney absorbed dose and no subacute grade 3 or 4 nephrotoxicity [20]. Long-term follow-up by other groups have also demonstrated low rates of nephrotoxicity from ^177^Lu when used with amino acids [11]. Subacute grade 3 or 4 haematological toxicities have been reported to occur in up to 11% of patients but generally recovered spontaneously; therefore, would not be considered dose-limiting. The incidence for therapy-related myeloid neoplasms (t-MN) including myelodysplasia (MDS) or acute leukaemia (AL) ranges from 1 to 4.8%. Although the underlying mechanisms remain poorly understood, these are not clearly related to cumulative administered activity [21].

In this study, we aim to perform an analysis to assess whether tumour dosimetry is independently predictive of response and prognosis in GEP NEN patients treated with PRRT. Specifically, we aim to investigate whether there is a tumour radiation dose-response relationship on ^68^ Ga DOTATATE (GaTate) positron emission tomography (PET) and RECIST 1.1 (Response Evaluation Criteria in Solid Tumours 1.1) on computed tomography (CT) [22]. We also aim to explore whether there are other predictive and prognostic factors based on disease characteristics, imaging biomarkers, treatment, and response assessment parameters in this very heterogenous disease.

## Material and methods

### Patients

All patients with unresectable GEP NEN who received induction PRRT with LuTate from July 2010 to January 2019 with a pre-treatment GaTate PET/CT, 24-h post-therapy Q-SPECT/CT, and 3-month post- PRRT GaTate PET/CT follow up were included. Patients with diffuse involvement of entire liver where lesion dosimetry was not technically feasible were not included. This study was approved by Human Research Ethics Committee (HREC) at Peter MacCallum Cancer Centre (LNR/60229) including a waiver for the requirement of consent due to the retrospective nature of the study.

### PET/CT volumetric measurements and RECIST 1.1 measurements

Molecular Imaging Tumour Volume of SSR (MITV_SSR_) and ^18^F Fluoro-Deoxy-Glucose (FDG) (MITV_FDG_) as well as the volume-intensity product (VIP) derived from multiplying the MITV with the SUVmean for each tracer, first described as total lesion glycolysis (TLG) for FDG, were measured. We prefer the derived parameters, VIP_SSR_ and VIP_FDG_, to harmonize nomenclature across the multiple tracers now available for assessing tumour biology. VIP parameters were measured on pre- PRRT GaTate and ^18^F FDG PET/CT (when available), respectively. Quantitative measurements were performed with a semi-automated workflow created on MIM software (MIM 6.9.4; MIM software, Cleveland, OH, USA) with segmentation based on 1.5 liver SUVmean + 2SD. RECIST 1.1 measurements were made on the low dose CT component of the GaTate PET/CT or, when available, on diagnostic contrast-enhanced CT (ceCT) [22].

### Treatments

LuTate was administered as per our institutional guidelines, with a standard administered activity of 8 GBq per cycle being individualised based on disease burden, renal function, and marrow toxicity from previous cycle/s. Specifically, administered activity was increased in patients with a large disease burden (which was largely a consensus opinion at weekly multidisciplinary meetings but broadly based on the presence of > 20 lesions or individual lesions > 5 cm) and reduced in patients with significantly impaired renal function (GFR < 60 ml/min), or who had demonstrated haematological toxicity with a prior cycle of PRRT, particularly if there had been incomplete recovery. Renal protective amino acid infusion (25 g/L lysine plus 25 gr/L arginine) was administered with each cycle, starting 30 min before LuTate administration, and continued for 2–4 h with longer infusions used for patients with impaired renal function [23]. RSC was administered mostly from the second cycle onwards if there was no contraindication. RSC protocols varied but typically included oral capecitabine alone or in combination with temozolomide.

### Dosimetry measurements

**Fig. 1:**
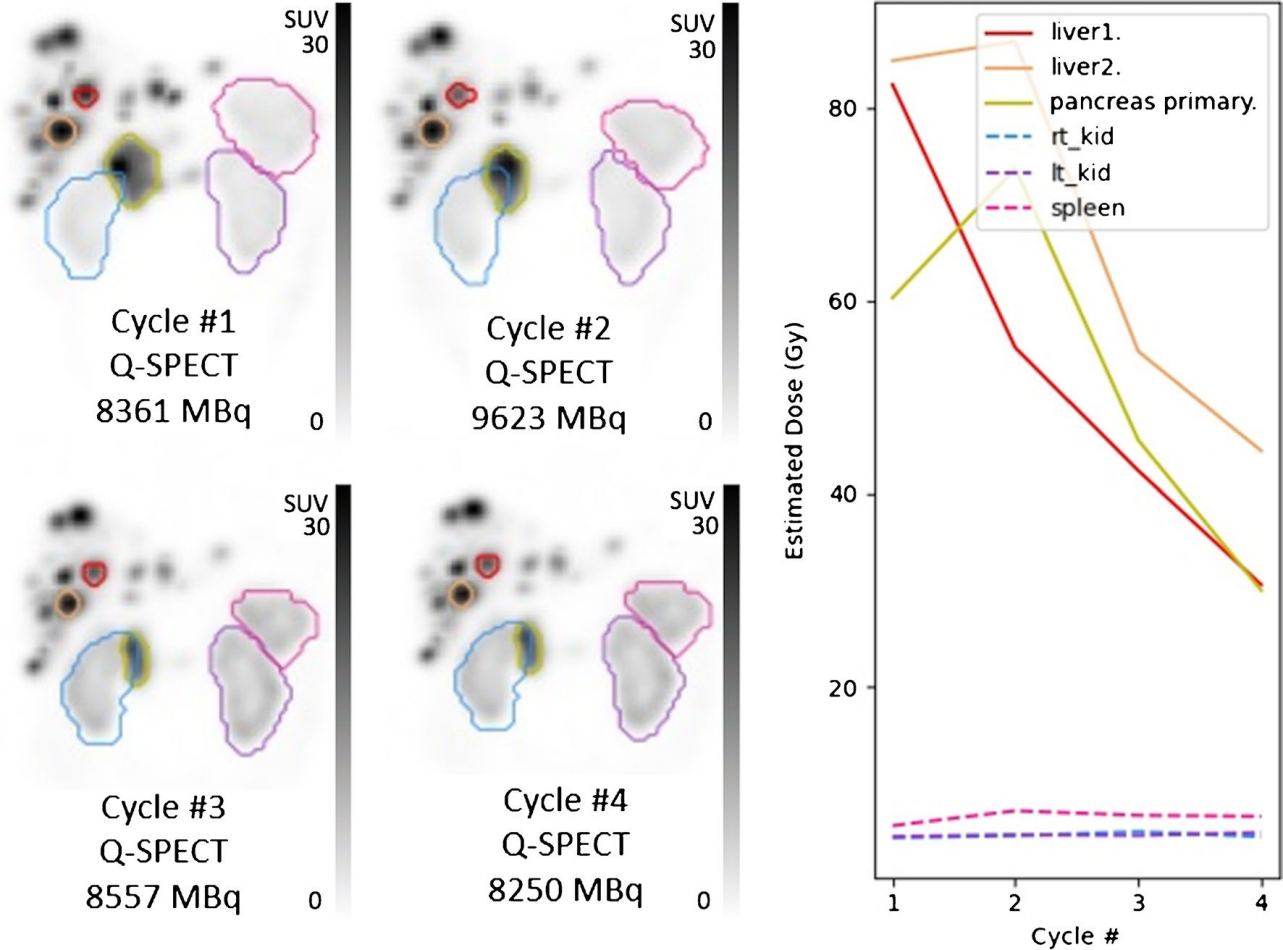
An example of STP dosimetry of lesions and physiological organs on 24-h Q-SPECT/CT. Maximal Intensity Projection (MIP) of post therapy Q-SPECTs after cycles 1–4 along with administered activities are displayed. In this case, liver 2 lesion was the most avid “index” lesion, and the pancreatic head primary was the “measurable lesion”

Single time-point (STP) dosimetry measurements of the most avid (index) lesion, combined lesions (measurable and non-measurable), and measurable lesions (up to 5, maximum 2 per organ) were performed on 24-h post PRRT Q-SPECT/CT for each cycle using population-based pharmacokinetic modelling (Fig. 1). Data to derive single timepoint dose factors based on representative population pharmacokinetic data for NEN [16] and the tabulated coefficients are provided in the Supplementary Material.

### Statistical methods

Descriptive statistics to summarise clinical data were reported in the form of means, medians, standard deviations, and ranges for quantitative variables. Categorical variables were reported as counts and percentages. Spearman correlation was used to measure the correlation between cumulative lesion tumour dosimetry and change in MITV_SSR_. The association of cumulative lesion dosimetry and prognostic factors with response was assessed using logistic regression. Logistic regression was also used to assess the relationship between dosimetry to physiologic organs with Grade 3 or 4 haematological toxicity. The analysis of prognostic factors for OS was performed using Cox proportional hazard models. Linear regression was used to assess and the analysis of predictive factors for MITV_SSR_. No adjustment for multiplicity was performed.

## Results

**Fig. 2:**
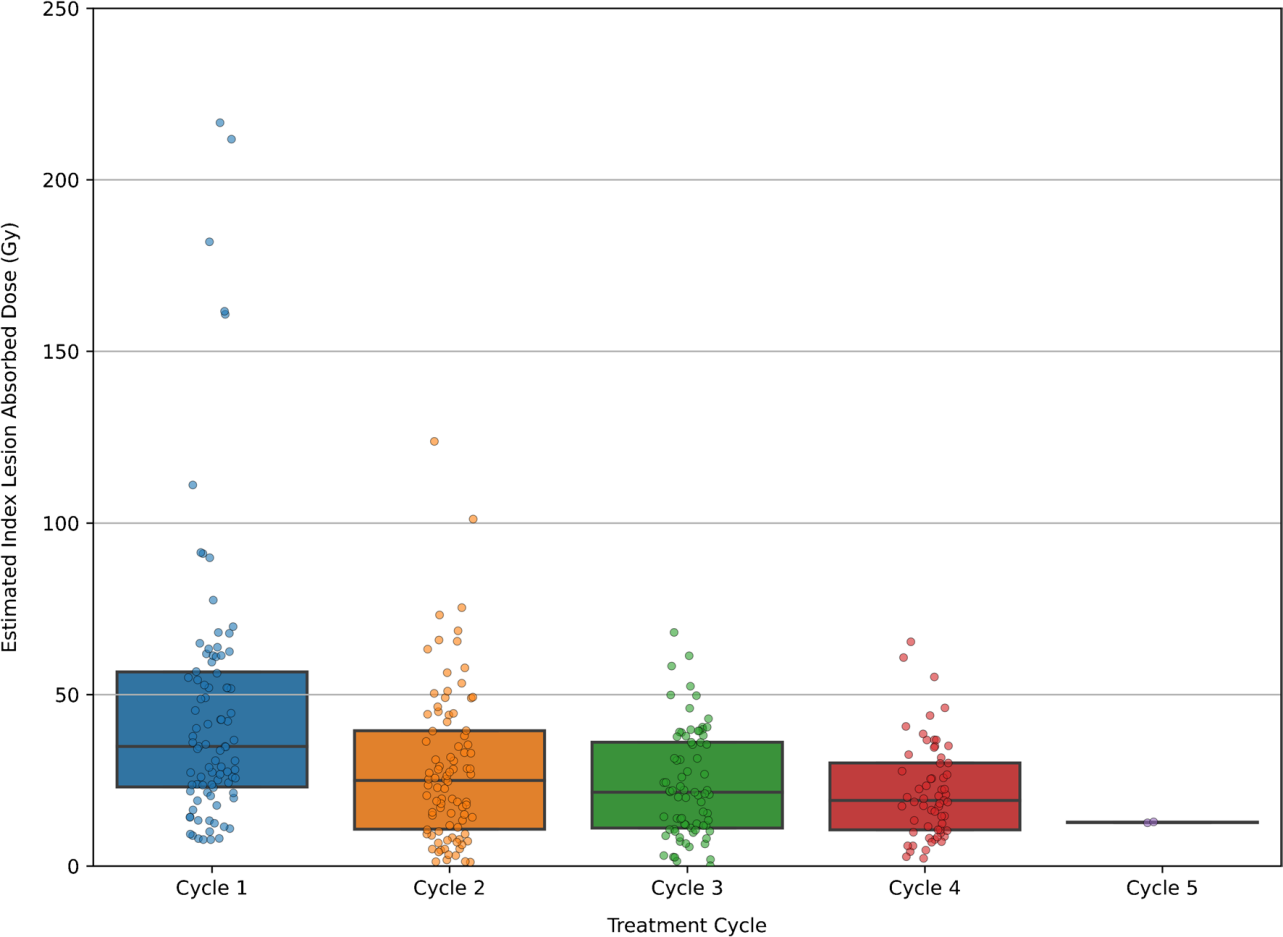
Index lesion estimated radiation absorbed dose (Gy) with different cycles of LuTate therapy

A total of 90 patients were included. The median age at the time of first PRRT cycle was 66, 52% were female. Primary site was most often small bowel (44%) or pancreas (40%). Most patients were G2 (61%) with a relatively equal distribution of G1 and G3 (19% and 20%, respectively) and a small number of patients with unknown grade (*n =* 9). Consistent with institutional policy that recommends ^18^F FDG PET/CT for G2 with Ki-67 > 10% and all G3 patients, or if progression has occurred over < 6 months in all other G1-2 cases, this scan had been performed in 63% of patients at baseline; 43% of which were positive and required to be concordant with GaTate to maintain eligibility. Four cycles of PRRT were administered to 63% of patients (range 2–5) with mean cumulative activity of 27.4 GBq (range 11.8–52.5 GBq) and mean per cycle activity of 7.6 GBq (range 3–12.3 GBq). 68% of patients (18% G1, 52% G2, 21% G3, 9% unknown grade) received RSC (mean age 60.3 vs 70.6 years in whom did not receive) with at least one cycle of PRRT (18% with one, 18% with two, 51% with three and 13% with four cycles). Capecitabine was used in 74%, capecitabine/temozolomide in 19% and 5-Fluro-uracil (5FU) in 7%. Mean cycle 1 and cumulative radiation dose for index lesion, combined lesions and measurable lesions were 46 Gy and 110 Gy, 33 Gy and 87 Gy, 34 Gy and 88 Gy, respectively. Mean per cycle radiation dose to the index lesion was 46.3 Gy, 28.4 Gy, 23.7 Gy, 22 Gy and 12.8 Gy for cycles 1 to 5 (where applicable), respectively (range 0.1–216.6 Gy). Table 1 provides a summary of patient’s characteristics and dosimetry measurements. Among patients receiving four treatment cycles (*n =* 59), the average fraction of index lesion cumulative dose delivered in the first treatment was 35.9% with a reduction in dose to 25.8%, 20.2%, and 18% at cycles 2–4, respectively. Estimated radiation absorbed dose to the index lesion with standard deviation bars for different cycles of LuTate therapy has bee provided in Fig. 2.

**Table 1:** Summary of patient’s characteristics and dosimetry measurements

	Total (*n =* 90)
Gender, *n* (%)	
Female	47 (52%)
Male	43 (48%)
Age, years	
Mean (SD)	64 (13)
Median [range]	66 [16–87]
IQR	56–71
Primary site, n (%)	
Appendix	1 (1%)
Caecum	2 (2%)
Gastric	1 (1%)
Pancreas	35 (40%)
Pancreas and Gastric	1 (1%)
Rectum	4 (5%)
Retroperitoneum	1 (1%)
Sacral or presacral	3 (3%)
Sigmoid	1 (1%)
Small bowel	39 (44%)
Unknown	2
Tumour grading, n (%)	
G1	15 (19%)
G2	50 (61%)
G3	16 (20%)
Unknown	9
Number of Lu-177 cycles, n (%)	
2	12 (13%)
3	17 (19%)
4	57 (63%)
5	4 (4%)
Cumulative Lu-177 activity, GBq	
Mean (SD)	27.4 (7.8)
Median [range]	28.4 [11.8–52.5]
IQR	22.4–32.2
Radiosensitising chemotherapy (RSC), n (%)	
No	29 (32%)
Yes	61 (68%)
Number of cycles with RSC, n (%)	
1	11 (18%)
2	11 (18%)
3	31 (51%)
4	8 (13%)
Baseline phenotype, n (%)	
GaTate vol greater than FDG	31 (36%)
GaTate vol same as FDG	6 (7%)
GaTate + and FDG-	20 (23%)
No FDG	33 (34%)
Index lesion cycle 1 radiation dose, Gy	
Mean (SD)	46 (41)
Median [range]	35 [8–217]
IQR	23–57
Index lesion cumulative radiation dose, Gy	
Mean (SD)	110 (78)
Median [range]	88 [11–380]
IQR	56–152
Measurable lesion cycle 1 Radiation Dose, Gy	
Mean (SD)	34 (24)
Median [range]	29 [5–135]
IQR	18–47
Measurable lesion cumulative Radiation Dose, Gy	
Mean (SD)	88 (56)
Median [range]	77 [8–244]
IQR	47–116
Combined lesions cycle 1 Radiation Dose, Gy	
Mean (SD)	33 (24)
Median [range]	27 [5–135]
IQR	17–45
Combined lesions cumulative radiation dose, Gy	
Mean (SD)	87 (57)
Median [range]	74 [8–238]
IQR	45–117

### MITV_SSR_ change

The median relative change in MITV_SSR_ post-PRRT was -63% with interquartile range from -84 to -29 (IQR: -84 to -29%). Table 2 shows the summary of MITV_SSR_ characteristics.

**Table 2:** Summary of MITV_SSR_ Characteristic

	Total (*n =* 90)
Baseline MITV_SSR_, ml	
Mean (SD)	253 (364)
Median [range]	94 [1–1661]
IQR	35–271
Post PRRT MITV_SSR_, ml	
Mean (SD)	128 (299)
Median [range]	34 [0–2382]
IQR	8–127
Missing	1
Relative change in MITV_SSR_, %	
Mean (SD)	-42 (78)
Median [range]	-63 [-100–408]
IQR	-84–-29

**Fig. 3:**
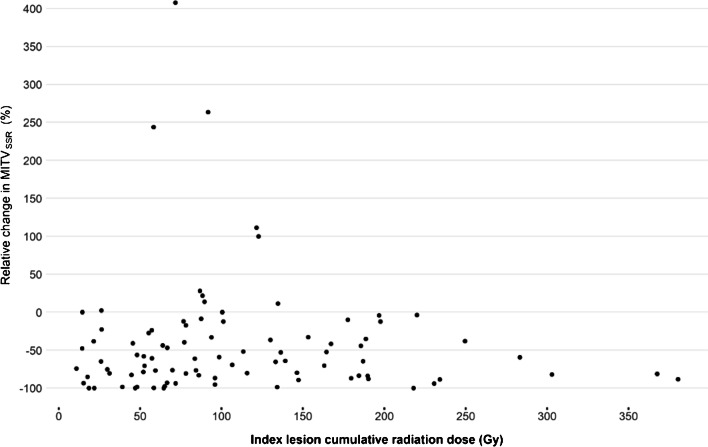
Index lesion cumulative radiation dose and relative change in MITV_SSR_

No correlation was found between index lesion radiation dose and change of MITV_SSR_ as a measure of total disease volume. The Spearman correlation between the index lesion cumulative radiation dose and relative change in MITV_SSR_ was 0.06 (*p =* 0.58). The scatter plot is provided below (Fig. 3). A linear regression was fit to assess the effect of index lesion cumulative radiation dose adjusting by tumour grade and RSC and splines were used to assess non-linearity. There was no evidence of non-linearity from the scatter plot or splines. The estimated relative change in MITV_SSR_ by index lesion cumulative radiation dose was 5.6 (95% CI: -1.6, 12.8) per 10 Gy increase, *p =* 0.133.

**Fig. 4:**
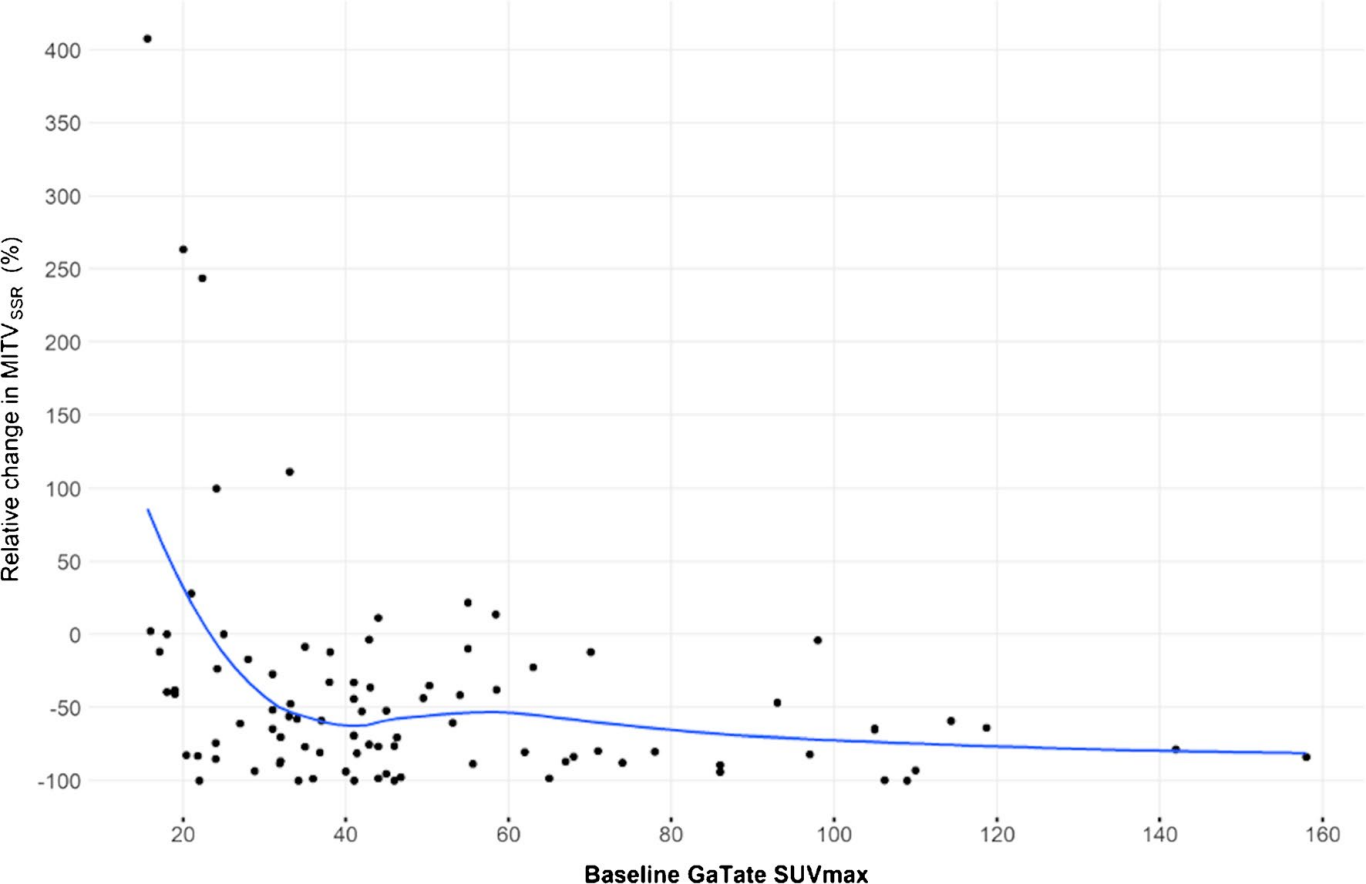
GaTate SUVmax and relative change in MITV_SSR_

The effect of baseline GaTate SUVmax on the relative reduction in MITV_SSR_ from baseline to 3 months follow-up was assessed, adjusting for tumour grade and RSC. There was evidence of non-linearity for baseline GaTate SUVmax when the model was fitted using spline. A scatter plot was generated with the solid line representing the non-linear association (spline). The results suggest reduction in MITV_SSR_ as baseline SUVmax increases up to approximately 35 with no relationship afterwards. However, this may be driven by the very large relative increase in MITV_SSR_ in some patients (outliers) with low baseline SUV_max_ (Fig. 4).

### RECIST 1.1 response

Of the 89 patients with RECIST response data available, there were 21 (24%) partial response (PR), 62 (70%) stable disease (SD) and 6 (7%) progressive disease (PD). Table 3 shows the prognostic factors for response (dichotomised as responders or non-responders) which shows association between all dosimetry variables and response. The odds ratio (OR) for measurable lesion cycle 1 radiation dose on response adjusted by tumour grade and RSC was 1.5 per 10 Gy increase (95% CI: 1.1–2.0), *p =* 0.002. Pancreatic primary was also predictive of response when compared to small bowel with OR 0.1 (95% CI: 0.0–0.5), *p =* 0.002, consistent with other literature [24].

**Table 3:** Prognostic factors for response

Variable	Level	OR (95% CI)	*p*-value
Tumour grading	G1 (ref)	-	-
	G2	4.5 (0.5, 38.2)	0.164
	G3	8.4 (0.9, 81.1)	0.066
Primary site	Pancreas (ref)	-	-
	Small bowel	0.1 (0.0, 0.5)	0.002
Baseline MITV_SSR_	Per 100 ml increase	1.0 (0.9, 1.2)	0.478
Baseline VIP_SSR_	Per 10 SUV x l increase	1.2 (0.6, 2.6)	0.592
Baseline GaTate SUVmax	Per 10 units increase	1.1 (1.0, 1.3)	0.130
Baseline MITV_FDG_	Per 10 ml increase	1.0 (1.0, 1.1)	0.432
Baseline VIP_FDG_	Per 100 SUV x ml increase	1.0 (0.9, 1.1)	0.569
Baseline FDG SUVmax	Per 5 units increase	1.2 (0.7, 2.0)	0.457
Baseline phenotype	GaTate vol greater than FDG (ref)	-	-
	GaTate vol same as FDG	1.4 (0.2, 9.4)	0.705
	GaTate + and FDG-	0.5 (0.1, 2.3)	0.411
	Unevaluable	1.0 (0.3, 3.3)	0.939
Cumulative Lu-177 activity	Per 10 GBq increase	1.3 (0.7, 2.5)	0.391
Index lesion cumulative radiation dose	Per 50 Gy increase	1.6 (1.1, 2.2)	0.006
Index lesion cycle 1 radiation dose	Per 10 Gy increase	1.3 (1.1, 1.6)	0.003
Measurable lesion cycle 1 Radiation Dose	Per 10 Gy increase	1.5 (1.2, 2.0)	0.001
CLD cumulative radiation dose	Per 50 Gy increase	1.6 (1.1, 2.5)	0.023
Radiosensitising chemotherapy	No (ref)	-	-
	Yes	1.7 (0.6, 5.4)	0.330
Number of radiosensitising chemotherapy cycles	Per unit increase	1.0 (0.6, 1.9)	0.901

### Overall survival

**Fig. 5:**
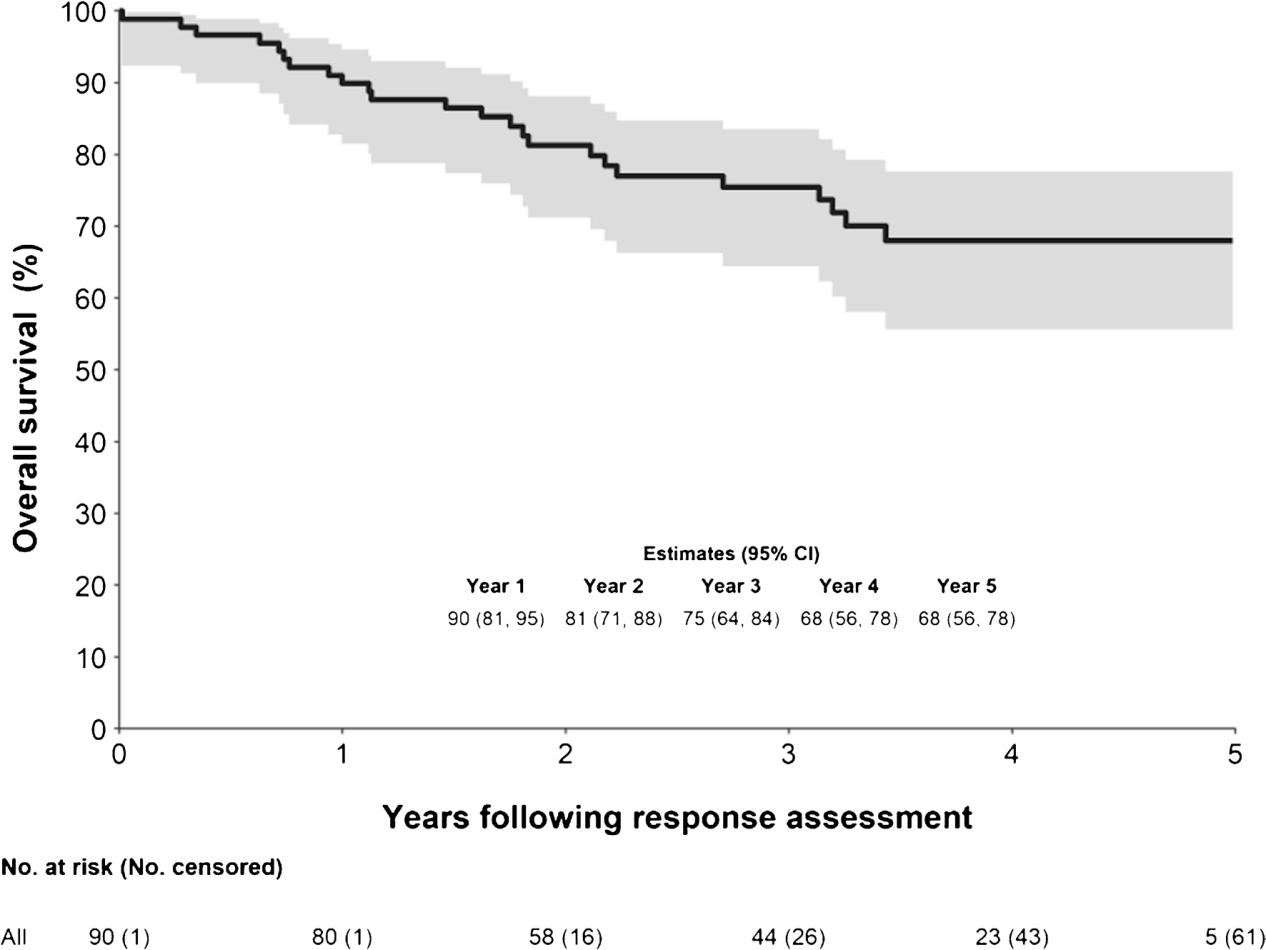
Overall survival curve

The OS at 5-years was 68% (95% CI: 56–78%) – (Fig. 5). Increasing baseline MITV_SSR_, increasing baseline FDG SUVmax, higher tumour grade and lack of RSC were suggestive of worse OS on univariable analysis (Table 4). When adjusted for tumour grade and RSC, there was no evidence that baseline MITV_SSR_ (HR [hazard ratio] 1.1 per 100 mL [95% CI: 1.0–1.2], *p =* 0.128) and relative change in MITV_SSR_ (HR = 1.0 per 20% increase [1.0- 1.1], *p =* 0.223) were associated with OS. It should be noted that, subject to contraindications, patients with higher MITV_SSR_ would have tended to be given a higher administered activity for at least cycle 1.

**Table 4:** Univariable analysis of prognostic factors for OS

Variable	Level	*N*	Events	HR (95% CI)	*p*-value
Tumour grading	G1	15	0	ref	0.036
	G2	50	14	Not estimable	
	G3	16	7	Not estimable	
Primary site	Pancreas	35	9	ref	0.665
	Small bowel	39	10	1.2 (0.5, 3.1)	
Baseline MITV_SSR_	Per 100 ml increase	90	25	1.1 (1.0, 1.2)	0.036
Baseline VIP_SSR_	Per 10 SUV x l increase	90	25	1.4 (0.8, 2.3)	0.239
Baseline GaTate SUVmax	Per 10 units increase	90	25	0.9 (0.8, 1.1)	0.251
Baseline MITV_FDG_	Per 10 ml increase	57	16	1.1 (1.0, 1.2)	0.130
Baseline VIP_FDG_	Per 100 SUV x ml increase	57	16	1.1 (1.0, 1.3)	0.090
Baseline FDG SUVmax	Per 5 units increase	56	16	1.8 (1.1, 2.8)	0.010
Baseline phenotype	GaTate vol greater than FDG	31	11	ref	0.242
	GaTate vol same as FDG	6	2	1.1 (0.2, 4.9)	
	GaTate + and FDG-	20	3	0.3 (0.1, 1.1)	
	Unevaluable	33	7	0.6 (0.2, 1.5)	
Cumulative Lu-177 activity	Per 10 GBq increase	90	25	1.2 (0.7, 2.0)	0.450
Index lesion cumulative radiation dose	Per 50 Gy increase	90	25	0.9 (0.7, 1.1)	0.275
Index lesion cycle 1 radiation dose	Per 10 Gy increase	90	25	0.9 (0.8, 1.1)	0.293
Measurable lesion cycle 1 Radiation Dose	Per 10 Gy increase	90	25	0.8 (0.7, 1.0)	0.108
CLD cumulative radiation dose	Per 50 Gy increase	90	25	0.8 (0.6, 1.2)	0.271
Radiosensitising chemotherapy	No	29	12	ref	0.012
	Yes	61	13	0.4 (0.2, 0.8)	
Number of radiosensitising chemotherapy cycles	Per unit increase	61	13	1.3 (0.7, 2.5)	0.368
Relative change in MITV_SSR_	Per 20% increase	90	25	1.1 (1.0, 1.1)	0.136
Relative change in MITV_FDG_	Per 20% increase	27	10	1.0 (1.0, 1.0)	0.748
RECIST response	Responded	21	4	ref	0.331

### Toxicity

There were no G3 or G4 renal toxicity within 3 months post PRRT. During PRRT cycles and the follow-up period, 11% (10/90) of patients developed G3 or G4 marrow toxicity; 6% (5/90) developed myelodysplastic syndrome (MDS) confirmed with bone marrow biopsy. One MDS patient (1%) progressed to AML. Cycle 1 radiation dose to spleen (OR = 1.23 per Gy [95% CI: 0.98–1.55}, *p =* 0.079) and cumulative spleen radiation dose (OR = 1.00 [95% CI: 0.92–1.08[, *p =* 0.979] were not associated with haematological toxicity.

## Discussion

### Absorbed dose in organ/tissue and tumour

Multiple time-point (MTP) dosimetry is highly demanding on patients and departments and often not practical in day-to-day clinical practice. This has resulted in a paradigm shift with a number of studies assessing STP dosimetry using population-based pharmacokinetic modelling [25–29]. Though the majority of dose estimates with STP dosimetry are close to true value, the main risk is large underestimation of time-integrated activity in some cases [18]. This is more relevant to normal tissue absorbed dose estimates as clearance of activity from these tissues is more variable than clearance from tumour which has high retention [17].

A small number of studies to date have tried to establish a dose-response relationship between the absorbed dose to the tumour during LuTate cycles and response in NEN. However, a consensus target absorbed dose for an effective therapy is yet to be defined. A STP dosimetry study of 24 lesions in 24 patients with metastatic pancreatic NEN treated with repeated cycles of LuTate demonstrated a significant correlation between the absorbed dose and tumour size reduction of 0.64 for tumours larger than 2.2 cm and 0.91 for a subgroup of tumours larger than 4.0 cm [14]. A MTP dosimetry study of a single lesion in 25 patients with metastatic pancreatic NEN and 23 patients with small bowel NEN treated with LuTate also demonstrated a dose-response relationship between the absorbed dose and shrinkage, which was more pronounced with pancreatic NENs [30]. In comparison to these studies, our study had the advantage of a larger patient cohort and analysis of multiple lesions in each patient on subsequent cycles. We were able to demonstrate association between radiation absorbed dose to the tumour (index lesion, combined lesions, and measurable lesions), both at cycle 1 and cumulatively with radiologic response, which is congruent with the limited available literature.

Another important observation from this study, is that the mean absorbed dose to the tumour throughout the cycles was not equally distributed. Index lesion, measurable lesions, and combined lesions radiation-absorbed doses at 1^st^ cycle were between 38–42% of the mean cumulative radiation-absorbed dose throughout all cycles (median 4). This finding will further support the notion of personalised prospective dosimetry and administering the highest tolerated activity with the first and second cycles to optimise response. Our empiric approach of modifying administered activity based on baseline imaging characteristics and post-treatment dosimetry estimates may have affected the ability to discern an impact of cumulative radiation dose on long-term survival. Diagnostic and therapeutic radionuclide pairs such as ^64^Cu/^67^Cu-SARTATE can potentially be used to achieve the ultimate goal of performing personalised prospective dosimetry [31].

### Response and overall survival

**Fig. 6:**
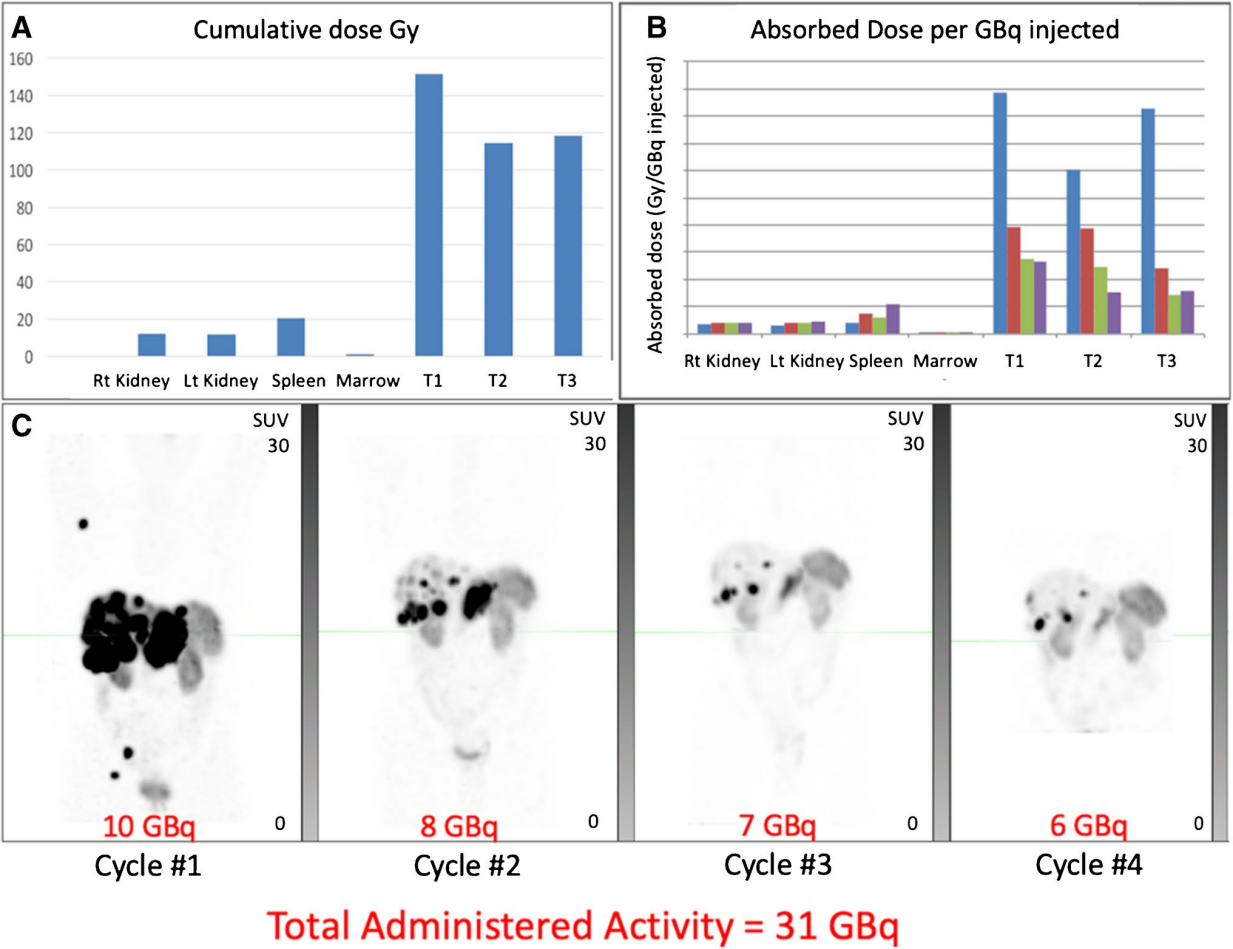
PRRT response in highly responsive NET. **A** Cumulative radiation dose (Gy) to physiological organs and three tumour lesions in the liver. **B** Absorbed dose (Gy) per GBq injected activity in physiological organs and three tumour lesions in the liver at cycles 1–4 (blue, red, green, and purple bars, respectively). The renal and spleen dose is generally lower for the first couple of cycles than later (although both are relatively stable during treatment) whereas tumour dose decreases and therefore therapeutic index is highest early. **C** MIP images of Q-SPECT 24-h post cycles 1–4 of LuTate therapy along with administered activity, totalling 31 GBq in 4 cycles

**Fig. 7:**
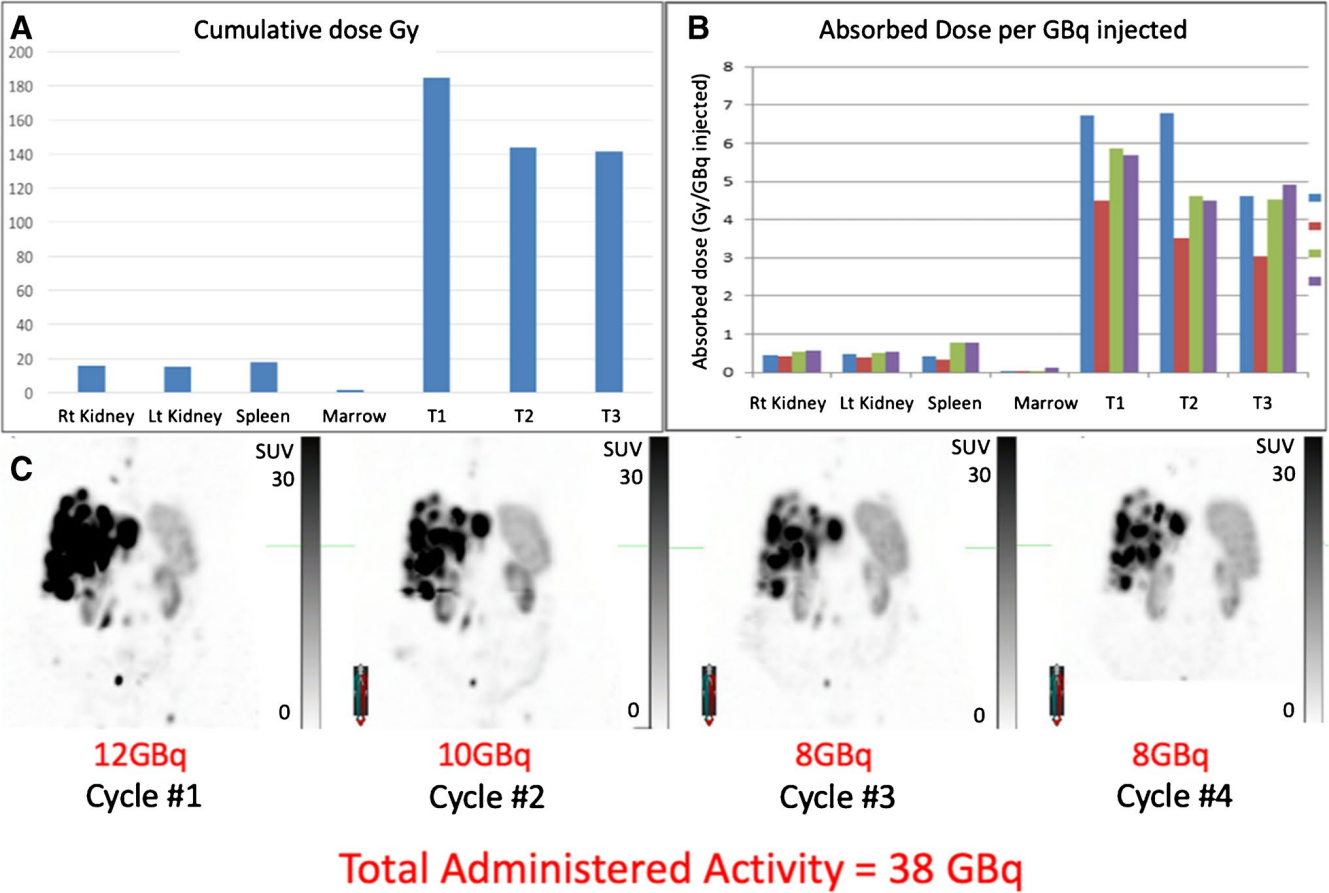
PRRT response in less responsive NET. **A** Cumulative radiation dose (Gy) to physiological organs and three tumour lesions in the liver. **B** Absorbed dose (Gy) per GBq injected activity in physiological organs and three tumour lesions in the liver at cycles 1–4 (blue, red, green, and purple bars, respectively). The renal and spleen dose is generally lower for the first couple of cycles than later (although both are relatively stable during treatment) whereas tumour dose decreases and therefore therapeutic index is highest early. **C** MIP images of Q-SPECT 24-h post cycles 1–4 of LuTate therapy along with administered activity, totalling 38 GBq in 4 cycles

Apart from absorbed dose to tumour, no other factors were strongly associated with radiologic response in our study. Interestingly, radiation dose to tumour did not correlate strongly with a change in MITV_SSR_, where some patients who received relatively lower tumour absorbed doses also had large reduction in MITV_SSR_, indicating that other biological factors, such as radiosensitivity of the tumour may impact response. We have, for example, found that metastatic rectal NET seems to be particularly responsive to PRRT [32]. Additionally, this suggests that the absorbed dose to the total disease volume, potentially granularly by individual lesion, should be considered to best predict overall response. Further, baseline GaTate SUVmax (as marker of tumor SSR uptake) may have a non-linear relationship with the relative change in MITV_SSR_ at 3 months post PRRT, possibly reflecting the importance of other factors such as radiosensitivity of disease at an individual level. While it is intuitive that high uptake in individual tumour sites should deliver significantly more radiation to these sites than those with lower uptake, and therefore also respond more rapidly, cumulative absorbed dose over subsequent cycles may be impacted by this response. As illustrated in a highly responsive case (Fig. 6) radiation doses to target lesions can reduce rapidly, which is contrasted with a less responsive case (Fig. 7) in which radiation doses were more evenly spread across all cycles. In both cases, the cumulative absorbed dose of target lesions was similar but the response in terms of MITV_SSR_ was markedly different.

In terms of prognosis, factors including increasing baseline MITV_SSR_ (a marker of tumour burden), baseline FDG SUVmax (reflecting metabolic activity), higher tumour grade and lack of RSC were suggestive of worse OS on univariable analysis. However, although it also appears intuitive that baseline MITV_SSR_ or relative change in MITV_SSR_ should be associated with OS, our multivariable analysis was not able to confirm this. This may be due to the small number of events (deaths) in our cohort which limits the power of the analysis, indicating the need for a larger study to properly address these questions. Tumour radiation dose or RECIST 1.1 response were also not associated with OS in this study. Possible explanations for these observations are due to biological factors: more indolent tumours may get to a larger size before coming to clinical attention but are likely to have a lower response to PRRT due to a combination of their low proliferative fraction and greater likelihood of hypoxia in large lesions. The lower objective response rate in small intestinal than pancreatic NET but lack of difference in OS would support the importance of intrinsic tumour biology to long-term outcomes. These observations are similar to those found with the use of chemotherapy in the NORDIC series wherein objective response was significantly lower in those with a Ki-67 < 55% but OS was significantly longer [33]. Accordingly, the response from PRRT may be less durable in higher grade disease but proportionally greater than that seen in lower grade NET.

### Increasing therapeutic effect of PRRT

Augmentation of therapeutic effect is another principle of radionuclide therapy in addition to imaging phenotyping, predictive dosimetry and post therapy assessment of radiation absorbed dose [34]. Preclinical models have shown an increase in double stranded DNA damage with addition of poly ADP-ribose polymerase (PARP) inhibitors to PRRT, increasing its efficacy [35]. A phase 1, single arm, single centre study to evaluate the safety and tolerability of PARP inhibitor (talazoparib) in combination with LuTate in patients with metastatic NET is currently underway [36]. In our series, 68% of patients received RSC with at least one cycle of PRRT. Lack of RSC was an adverse predictor of OS on univariate analysis; however, this may be due a selection bias as generally younger patients were selected for this combination and older patients with contraindications were excluded. Conversely, at our institution, chemotherapy is generally advocated for high G2/G3 patients or those with FDG-avid NET. Both these features are known to have adverse prognostic influence and therefore support the benefit of RSC. Confining RSC to higher risk patients may be justified by the observation of a slightly higher rate of MDS in this cohort (6%) compared to (4%) in Bergsma et.al series [37], which could reflect the synergistic marrow toxicity of PRRT and RSC, or pre-existing biological or genetic susceptibility to the development of therapy related myeloid neoplasm (t-MN). No association between splenic radiation dose and haematologic toxicity was demonstrated in this study. However, this combination did not seem to adversely affect kidney function with no G3 or G4 nephrotoxicity observed up to 3 months post completion of PRRT, similar to Bergsma et al. [20].

### Limitations

The limitations of our study were its retrospective nature and the relatively small number of patients with rather heterogenous malignancies of different primary sites. Our personalised approach in prescribing the administered activity may have also impacted the lack of correlation between the change in MITV_SSR_ and OS. Nevertheless, these results would be of interest as they are hypothesis generating and exploratory.

## Conclusion

Radiation absorbed doses of lesions, both at cycle 1 and cumulatively during PRRT cycles were predictive of radiologic response, indicating a dose-response relationship in patients with NEN. However, other factors including biology appear to be more important to OS indicating heterogeneity of neuroendocrine tumours. There appears to be substantial opportunity to safely increase radiation dose to lesions early during PRRT cycles with evidence that the higher proportion of cumulative dose to disease sites is delivered with first treatment. The future research direction should be to leverage the high therapeutic index in the earlier stages of treatment to increase radiation dose to lesions without incremental toxicity, and development of personalised prospective dosimetry to improve overall outcomes.

## Supplementary information

Below is the link to the electronic supplementary material.

Supplementary file1 (DOCX 599 KB)

## Data Availability

All data supporting the findings of this study are available within the paper and its Supplementary Information.
