# Supplementary material for: The relationship between tumour dosimetry, response, and overall survival in patients with unresectable Neuroendocrine Neoplasms (NEN) treated with 177Lu DOTATATE (LuTate)
Source: Eur J Nucl Med Mol Imaging. 2023 May 15;50(10):2997–3010. doi: 10.1007/s00259-023-06257-6 (PMC10382388; doi:10.1007/s00259-023-06257-6)
Supplement: Supplementary file 1 — Supplementary file1 (DOCX 599 KB) [file 259_2023_6257_MOESM1_ESM.docx]

# Supplementary Material

Methods to derive Single Timepoint Dose Factors to Tumour:

Serial SPECT/CT acquisitions were recorded at 4-, 24-, & 72-hours post-injection for twenty-eight instances of therapy with 177Lu-DOTA-octreotate for treatment of disseminated neuroendocrine neoplasms. Quantitation of SPECT images (Symbia T6, Siemens A.G., Darmstad, Germany) was performed by previously reported protocol involving attenuation, dead-time, & scatter correction [17]. For each treatment, images were aligned by sequential rigid and deformable registration and areas of disease were contoured by expert clinician. The mean activity concentration for each imaging timepoint was utilised to determine a three-phase exponential time-activity curves. The six parameters used to define the best fit curve were normalised by amplitude (preserving all rate information and relative contribution of each phase) to match the uptake at a single post-injection time point (Supplementary Figure 1). This allows estimation of time integrated activity for any intermediate time value.


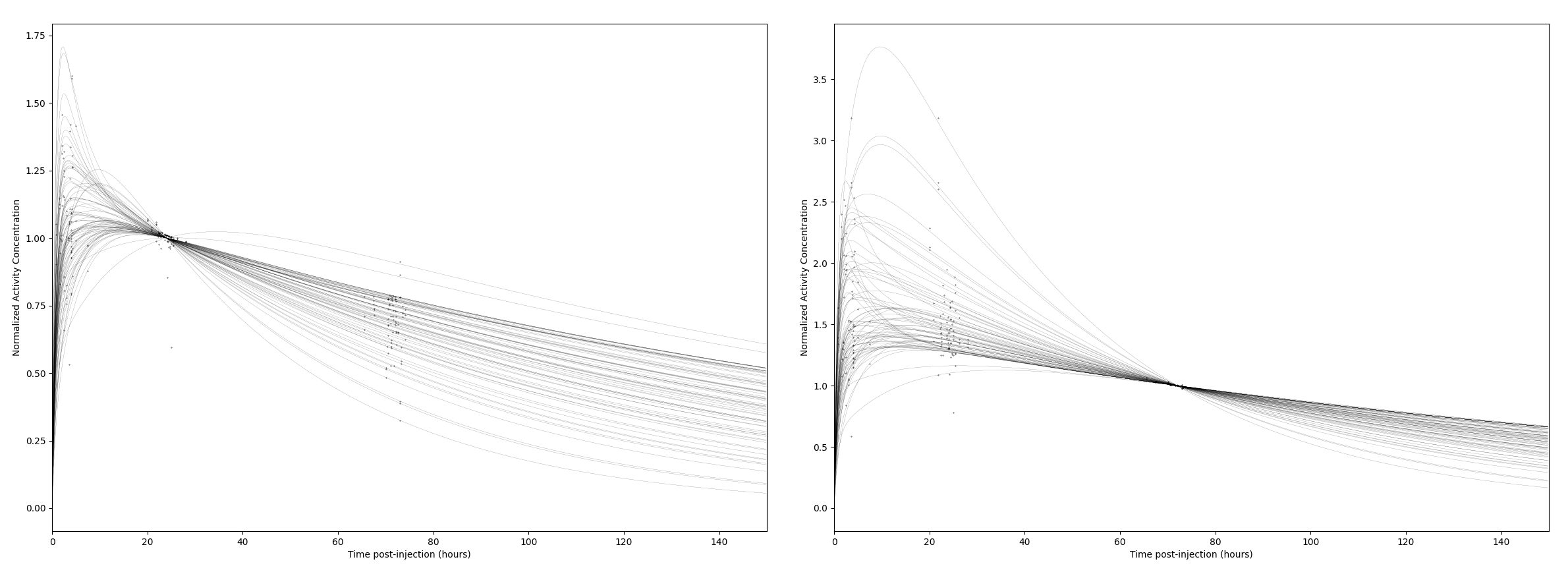


Supplementary Figure 1: Method of normalisation to a single post-injection time point to determine the mean and range of estimated cumulated time-integrated activity values according to the representative treatment cohort. For all curves the temporal parameters remain constant, while absolute activity scaling factors are applied to allow convergence at the desired post-injection time time-point as illustrated for times of 24 hours (left) and 72 hours (right) post-injection.

The mean and standard deviation of cumulated time-integrated activity concentration were recorded to determine a representative number of decays per unit volume and to characterise the uncertainty for the specified organ and imaging time point. Regional cumulated time-integrated activity concentration was converted to local radiation absorbed dose with a representative OLINDA sphere model dose factor of 8.7E-5 Gy/(kBq*h/ml). This coefficient is multiplied by the time-integral coefficient to determine a single timepoint dose factor to convert between measured activity concentration (kBq/ml) at a given imaging time and radiation absorbed dose (Gy) to tissue. The tabulated dose factors and relative uncertainty are indicated in Supplementary Figure 2. Population average dose coefficients and relative uncertainty according to standard deviation are reported in Supplementary Table 1.


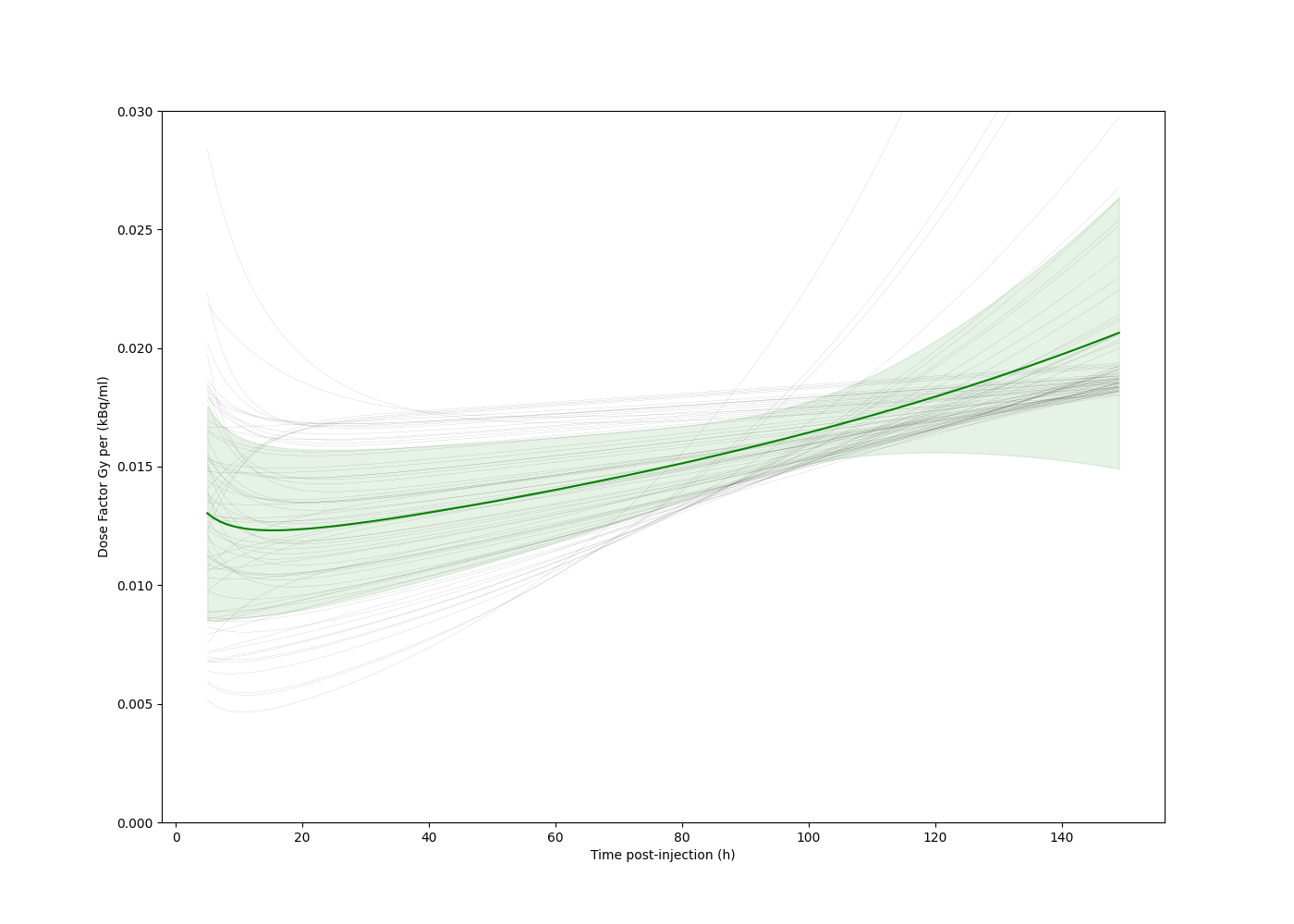


Supplementary Figure 2: Imaging time-dependent tumour dose factors (Gy per kBq/ml) factors derived for tumour based on sample population data. Predicted factors for individual lesions are indicated in fine grey lines while population average and standard deviation is shown with green and shaded area.

| Time post-injection (h) | Tumour Dose Factor (Gy*ml/kBq) | Population standard deviation σ (%) |
| --- | --- | --- |
| 5 | 0.013034 | 34.7% |
| 6 | 0.012832 | 33.6% |
| 7 | 0.012685 | 32.7% |
| 8 | 0.012575 | 32.0% |
| 9 | 0.012493 | 31.4% |
| 10 | 0.012431 | 30.8% |
| 11 | 0.012385 | 30.3% |
| 12 | 0.012353 | 29.9% |
| 13 | 0.012331 | 29.5% |
| 14 | 0.012318 | 29.2% |
| 15 | 0.012313 | 28.8% |
| 16 | 0.012314 | 28.5% |
| 17 | 0.01232 | 28.2% |
| 18 | 0.012331 | 27.8% |
| 19 | 0.012345 | 27.5% |
| 20 | 0.012363 | 27.2% |
| 21 | 0.012384 | 26.9% |
| 22 | 0.012407 | 26.6% |
| 23 | 0.012433 | 26.3% |
| 24 | 0.012461 | 26.0% |
| 25 | 0.01249 | 25.8% |
| 26 | 0.012521 | 25.5% |
| 27 | 0.012554 | 25.2% |
| 28 | 0.012587 | 24.9% |
| 29 | 0.012622 | 24.6% |
| 30 | 0.012658 | 24.3% |
| 31 | 0.012695 | 24.0% |
| 32 | 0.012733 | 23.8% |
| 33 | 0.012772 | 23.5% |
| 34 | 0.012812 | 23.2% |
| 35 | 0.012852 | 22.9% |
| 36 | 0.012893 | 22.6% |
| 37 | 0.012934 | 22.3% |
| 38 | 0.012976 | 22.1% |
| 39 | 0.013019 | 21.8% |
| 40 | 0.013062 | 21.5% |
| 41 | 0.013106 | 21.2% |
| 42 | 0.01315 | 20.9% |
| 43 | 0.013195 | 20.6% |
| 44 | 0.01324 | 20.4% |
| 45 | 0.013286 | 20.1% |
| 46 | 0.013332 | 19.8% |
| 47 | 0.013379 | 19.5% |
| 48 | 0.013426 | 19.2% |
| 49 | 0.013473 | 18.9% |
| 50 | 0.013521 | 18.7% |
| 51 | 0.013569 | 18.4% |
| 52 | 0.013618 | 18.1% |
| 53 | 0.013667 | 17.8% |
| 54 | 0.013716 | 17.5% |
| 55 | 0.013766 | 17.2% |
| 56 | 0.013816 | 16.9% |
| 57 | 0.013867 | 16.7% |
| 58 | 0.013918 | 16.4% |
| 59 | 0.013969 | 16.1% |
| 60 | 0.014021 | 15.8% |
| 61 | 0.014073 | 15.5% |
| 62 | 0.014126 | 15.2% |
| 63 | 0.014179 | 14.9% |
| 64 | 0.014232 | 14.7% |
| 65 | 0.014286 | 14.4% |
| 66 | 0.01434 | 14.1% |
| 67 | 0.014394 | 13.8% |
| 68 | 0.014449 | 13.5% |
| 69 | 0.014504 | 13.2% |
| 70 | 0.01456 | 13.0% |
| 71 | 0.014616 | 12.7% |
| 72 | 0.014673 | 12.4% |
| 73 | 0.01473 | 12.1% |
| 74 | 0.014787 | 11.9% |
| 75 | 0.014845 | 11.6% |
| 76 | 0.014903 | 11.3% |
| 77 | 0.014962 | 11.1% |
| 78 | 0.015021 | 10.8% |
| 79 | 0.01508 | 10.6% |
| 80 | 0.01514 | 10.3% |
| 81 | 0.0152 | 10.1% |
| 82 | 0.015261 | 9.9% |
| 83 | 0.015323 | 9.6% |
| 84 | 0.015384 | 9.4% |
| 85 | 0.015446 | 9.2% |
| 86 | 0.015509 | 9.0% |
| 87 | 0.015572 | 8.9% |
| 88 | 0.015636 | 8.7% |
| 89 | 0.0157 | 8.5% |
| 90 | 0.015764 | 8.4% |
| 91 | 0.015829 | 8.3% |
| 92 | 0.015895 | 8.1% |
| 93 | 0.015961 | 8.1% |
| 94 | 0.016027 | 8.0% |
| 95 | 0.016094 | 7.9% |
| 96 | 0.016162 | 7.9% |
| 97 | 0.01623 | 7.9% |
| 98 | 0.016298 | 7.9% |
| 99 | 0.016367 | 7.9% |
| 100 | 0.016437 | 8.0% |
| 101 | 0.016507 | 8.1% |
| 102 | 0.016578 | 8.2% |
| 103 | 0.016649 | 8.3% |
| 104 | 0.016721 | 8.4% |
| 105 | 0.016793 | 8.6% |
| 106 | 0.016866 | 8.8% |
| 107 | 0.01694 | 9.0% |
| 108 | 0.017014 | 9.2% |
| 109 | 0.017088 | 9.5% |
| 110 | 0.017164 | 9.7% |
| 111 | 0.01724 | 10.0% |
| 112 | 0.017316 | 10.3% |
| 113 | 0.017393 | 10.6% |
| 114 | 0.017471 | 10.9% |
| 115 | 0.017549 | 11.3% |
| 116 | 0.017628 | 11.6% |
| 117 | 0.017708 | 12.0% |
| 118 | 0.017788 | 12.3% |
| 119 | 0.017869 | 12.7% |
| 120 | 0.01795 | 13.1% |
